# Doxycycline Postexposure Prophylaxis and Bacterial Sexually Transmitted Infections Among Individuals Using HIV Preexposure Prophylaxis

**DOI:** 10.1001/jamainternmed.2024.7186

**Published:** 2025-01-06

**Authors:** Michael W. Traeger, Wendy A. Leyden, Jonathan E. Volk, Michael J. Silverberg, Michael A. Horberg, Teaniese L. Davis, Kenneth H. Mayer, Douglas S. Krakower, Jessica G. Young, Samuel M. Jenness, Julia L. Marcus

**Affiliations:** 1 Department of Population Medicine, Harvard Pilgrim Health Care Institute, Boston, Massachusetts; 2 Burnet Institute, Melbourne, Australia; 3 School of Public Health and Preventive Medicine, Monash University, Melbourne, Australia; 4 Division of Research, Kaiser Permanente Northern California, Pleasanton; 5 Department of Infectious Diseases, Kaiser Permanente San Francisco, San Francisco, California; 6 Department of Health Systems Science, Kaiser Permanente Bernard J. Tyson School of Medicine, Pasadena, California; 7 Departments of Epidemiology andBiostatistics, University of California, San Francisco; 8 Department of Medicine, University of California, San Francisco; 9 Kaiser Permanente Mid-Atlantic States, Mid-Atlantic Permanente Medical Group, Washington, District of Colombia; 10 Center for Research and Evaluation, Kaiser Permanente Georgia, Atlanta; 11 The Fenway Institute, Fenway Health, Boston, Massachusetts; 12 Harvard Medical School, Harvard University, Boston, Massachusetts; 13 Division of Infectious Diseases, Beth Israel Deaconess Medical Center, Boston, Massachusetts; 14 Department of Epidemiology, Rollins School of Public Health, Emory University, Atlanta, Georgia

## Abstract

**Importance:**

Doxycycline postexposure prophylaxis (doxyPEP) has been shown to decrease the incidence of bacterial sexually transmitted infections (STIs) among people assigned male sex at birth in clinical trials, but data from clinical practice are limited.

**Objective:**

To describe early uptake of doxyPEP and evaluate changes in STI incidence following doxyPEP initiation.

**Design, Setting, and Participants:**

This retrospective cohort study of adults (aged ≥18 years) dispensed HIV preexposure prophylaxis (PrEP) at Kaiser Permanente Northern California during November 1, 2022, to December 31, 2023, examined electronic health record data to compare HIV PrEP users dispensed and not dispensed doxyPEP and rates of bacterial STIs before and after starting doxyPEP. Individuals were followed up from their first recorded STI test on or after November 1, 2020, until December 31, 2023, or discontinuation of health plan membership.

**Exposure:**

Pharmacy dispensing data were used to define doxyPEP recipients.

**Main Outcomes and Measures:**

Demographic and clinical characteristics were compared between individuals dispensed and not dispensed doxyPEP. Primary outcomes were incident chlamydia, gonorrhea, or infectious syphilis measured as quarterly STI positivity (proportion of individuals testing positive at least once per quarter). Among doxyPEP recipients, rate ratios (RRs) compared mean quarterly STI positivity from 24 months before to 12 months after starting doxyPEP. In an exploratory analysis, STI trends were evaluated for the full cohort, stratified by receipt of doxyPEP.

**Results:**

Among 11 551 HIV PrEP users (mean [SD] age, 39.9 [12.1] years; 95.1% male), 2253 (19.5%) were dispensed doxyPEP, of whom 2228 (98.9%) were male and 1096 (48.6%) had an STI in the year before starting doxyPEP. Compared with individuals not dispensed doxyPEP, doxyPEP recipients were older (mean [SD] age, 40.4 [10.8] vs 39.8 [12.4] years; *P* = .04) and had used HIV PrEP longer (mean [SD], 4.2 [2.8] vs 3.4 [2.6] years; *P* < .001), and a higher proportion were commercially insured (2091 [92.8%] vs 8270 [88.9%]; *P* < .001). Among doxyPEP recipients, quarterly chlamydia positivity decreased from 9.6% (95% CI, 9.0%-10.3%) before starting doxyPEP to 2.0% (95% CI, 1.5%-2.6%) after starting doxyPEP (RR, 0.21; 95% CI, 0.16-0.27; *P* < .001), with significant declines for each anatomic site of infection. Quarterly gonorrhea positivity decreased from 10.2% (95% CI, 9.6%-10.9%) before starting doxyPEP to 9.0% (95% CI, 8.0%-10.1%) after starting doxyPEP (RR, 0.88; 95% CI, 0.77-1.00; *P* = .048); site-specific declines were significant for rectal (RR, 0.81; 95% CI, 0.67-0.97; *P* = .02) and urethral (RR, 0.56; 95% CI, 0.40-0.79; *P* = .001) gonorrhea, but not pharyngeal gonorrhea. Quarterly syphilis positivity decreased from 1.7% (95% CI, 1.4%-1.9%) before starting doxyPEP to 0.3% (95% CI, 0.2%-0.6%) after starting doxyPEP (RR, 0.20; 95% CI, 0.11-0.37; *P* < .001). Positivity for STIs remained stable in individuals not dispensed doxyPEP.

**Conclusions and Relevance:**

This study found that receipt of doxyPEP was associated with substantial declines in chlamydia and syphilis incidence and modest declines in urethral and rectal gonorrhea incidence among individuals using HIV PrEP. These findings suggest that doxyPEP may offer substantial benefits for reducing population-level STI transmission with broader implementation.

## Key Points

**Question:**

Is the receipt of doxycycline postexposure prophylaxis (doxyPEP) associated with declines in incidence of bacterial sexually transmitted infections (STIs) outside of clinical trials?

**Findings:**

In this cohort study of 11 551 individuals (95.1% male) receiving HIV preexposure prophylaxis in an integrated health system in California, 2253 were dispensed doxyPEP. Among doxyPEP recipients, quarterly STI positivity significantly declined from before to after starting doxyPEP by 79% for chlamydia, 80% for syphilis, and 12% for gonorrhea.

**Meaning:**

These findings suggest that doxyPEP may offer substantial benefits for decreasing population-level STI transmission with broader implementation.

## Teaser

This cohort study evaluates the early uptake of doxycycline postexposure prophylaxis (doxyPEP) and changes in sexually transmitted infection incidence following doxyPEP initiation.

## Introduction

In recent clinical trials, 200-mg doxycycline postexposure prophylaxis (doxyPEP) taken within 72 hours of sex has been shown to reduce the risk of bacterial sexually transmitted infections (STIs)—specifically chlamydia and syphilis, with mixed results for gonorrhea—among people assigned male sex at birth and either living with HIV or using HIV preexposure prophylaxis (PrEP).^1,2,3^ In October 2022, San Francisco, California, became the first US jurisdiction to issue guidance on doxyPEP provision.^4^ The Centers for Disease Control and Prevention released clinical guidelines in June 2024 recommending that clinicians counsel all gay, bisexual, and other men who have sex with men and transgender women with a bacterial STI diagnosis in the past 12 months about doxyPEP and offer doxyPEP through shared decision-making.^5^ Evidence of the prevention benefits of doxyPEP in clinical practice may help support implementation.

In this study, we describe scale-up of doxyPEP among HIV PrEP users in a large health care system in California. We had 3 objectives: (1) describe doxyPEP uptake, including characteristics of early adopters and patterns of use; (2) evaluate changes in bacterial STI incidence and testing from before to after doxyPEP initiation among doxyPEP recipients; and (3) compare trends in STI incidence before and after doxyPEP implementation between individuals dispensed and not dispensed doxyPEP.

## Methods

### Study Setting and Population

This retrospective cohort study included all adult (aged ≥18 years) Kaiser Permanente Northern California (KPNC) members dispensed HIV PrEP from November 1, 2022, through December 31, 2023, including members who were and were not dispensed doxyPEP. This study was approved by the Kaiser Permanente Northern California Institutional Review Board with a waiver of informed consent because the study used existing clinically derived data and involved no more than minimal risk. The study followed the Strengthening the Reporting of Observational Studies in Epidemiology (STROBE) reporting guideline.

Kaiser Permanente Northern California is an integrated health care system delivering comprehensive medical services to 4.6 million members, with a diverse patient population that is demographically representative of insured people in the region.^6^ Beginning in November 2022, doxyPEP was offered to KPNC members accessing HIV PrEP care, which is delivered by dedicated HIV PrEP teams. Bacterial STI testing is recommended at least quarterly during HIV PrEP use. At KPNC, testing is available on a drop-in basis and as often as monthly for most HIV PrEP users.^7^ Bacterial STI testing recommendations did not change for HIV PrEP users who initiated doxyPEP.

Although outreach to offer doxyPEP varied by medical facility, prescribing was recommended across facilities for individuals assigned male sex at birth who in the past year had a bacterial STI and reported condomless sex with more than 1 partner. If individuals did not have a recent bacterial STI but were interested in doxyPEP, clinicians used shared decision-making. Decisions about doxyPEP prescribing were individualized for patients assigned female at birth, given limited data for this population. Individuals were followed up from their first recorded STI test on or after November 1, 2020, until December 31, 2023, or disenrollment from the health plan, whichever occurred first.

### Data Collection

We extracted data from KPNC’s electronic health records. Sociodemographic characteristics included age, self-reported race and ethnicity (Hispanic; non-Hispanic American Indian or Alaska Native, Asian, Black or African American, Native Hawaiian or Pacific Islander, White, or multiracial; and unknown), sex (female, male, unknown), and health insurance status. Data on race and ethnicity were extracted and analyzed to characterize the population receiving doxyPEP and potential disparities in prescribing. Pharmacy data were used to estimate time since first being dispensed HIV PrEP at KPNC.

Laboratory data included tests and results for chlamydia, gonorrhea, and syphilis. As part of the KPNC PrEP program, testing for urethral, pharyngeal, and rectal chlamydia and gonorrhea is conducted using dual nucleic acid amplification tests. Testing at each anatomic site is recommended based on sexual exposures. New cases of syphilis were determined from laboratory results based on the reverse sequence testing algorithm (eMethods in Supplement 1).^8^

We identified doxyPEP fills using dosing instructions from pharmacy data (ie, take 200 mg within 72 hours of sex) and internal diagnostic codes for doxyPEP. We excluded prescriptions with 2 or fewer 100-mg pills and prescriptions for which dosing instructions suggested indications other than STI prophylaxis.

### Outcomes

The primary outcomes were incident chlamydia, gonorrhea, or syphilis measured as quarterly STI positivity. We defined STI positivity as the proportion of individuals tested with at least 1 positive result for the respective STI during quarterly follow-up windows. We calculated quarterly STI positivity for each STI separately and by each anatomic site for chlamydia and gonorrhea.

We explored a secondary outcome of STI testing, measured as the proportion of individuals contributing follow-up time throughout a quarterly window who had at least 1 test for the respective STI during that window. We also calculated the mean number of STI tests per person per quarter among individuals tested at least once in the respective quarter.

### Statistical Analysis

We used descriptive statistics to compare characteristics of individuals dispensed and not dispensed doxyPEP. For individuals dispensed doxyPEP, we described median numbers of pills dispensed in the first doxyPEP fill, fills per person, and doses in possession per month.

To evaluate changes in STI incidence and testing, we conducted (1) a primary analysis comparing STI positivity and testing before and after initiating doxyPEP among individuals dispensed doxyPEP and (2) an exploratory analysis of trends in quarterly STI positivity by calendar time in the full cohort, including those both dispensed and not dispensed doxyPEP, before and after doxyPEP was implemented at KPNC.

In the primary analysis on STI incidence among individuals dispensed doxyPEP, we compared quarterly STI positivity in the 24 months before to 12 months after initiating doxyPEP. For the primary analysis, we chose a pre/post descriptive analytic design. Inclusion of a matched control group with imputed index dates was deemed unsuitable given the likelihood of time-varying confounding arising from the association between the outcome of interest (STI diagnosis) and probability of starting doxyPEP. We defined a baseline window for each individual as the period from 90 days before to 14 days after the first doxyPEP fill. We excluded STI diagnoses during this window to minimize potential bias from increased STI detection around the time of doxyPEP initiation (ie, increased likelihood of starting doxyPEP following a recent STI diagnosis). We used Poisson regression to estimate mean percent change in STI positivity per quarter (trend), with corresponding 95% CIs, separately for the periods before and after individuals initiated doxyPEP. We then used Poisson regression to compute rate ratios (RRs) representing the relative difference in mean quarterly STI positivity from the period before to the period after doxyPEP initiation by including a time-updated independent variable indicating whether an individual had initiated doxyPEP. In these models, the dependent variable was STI positivity (restricted to a maximum of 1 positive test per individual per quarter), with an independent continuous time covariate (quarter from baseline). Robust standard error calculations clustered by individual accounted for multiple observations per person. To explore the potential effect on our results of some doxyPEP recipients not having a full 12 months of post–doxyPEP initiation follow-up, we compared mean STI positivity in the 12 months before to 6 months after starting doxyPEP among those who were dispensed doxyPEP before June 30, 2023, and therefore had at least 6 months of follow-up after starting doxyPEP.

To assess potential changes in STI testing following doxyPEP initiation, we computed RRs comparing the mean proportion of individuals tested per quarter across 12 months before and 12 months after initiating doxyPEP. Among individuals with at least 6 months of follow-up after doxyPEP initiation, we computed the proportion with at least 1 STI test within that window.

We conducted 2 exploratory analyses to compare STI incidence before and after doxyPEP implementation between individuals dispensed and not dispensed doxyPEP. First, we used Poisson regression to estimate trends in quarterly STI positivity (mean percent change per quarter) during the pre-doxyPEP (quarter 1 [Q1] 2021 to Q3 2022) and post-doxyPEP (Q4 2022 to Q4 2023) implementation periods separately for the groups dispensed and not dispensed doxyPEP at least once during the study period. Second, for the post-doxyPEP implementation period, we computed RRs comparing quarterly STI positivity between doxyPEP recipients after they had initiated doxyPEP and those never dispensed doxyPEP using multivariable Poisson regression to adjust for age, calendar quarter, race and ethnicity, and type of insurance.

DoxyPEP implementation at non-KPNC sites in San Francisco following issuance of local doxyPEP guidelines in October 2022^4^ may have influenced population-level STI transmission. Therefore, we conducted a post hoc analysis exploring cohort-level trends in STI positivity disaggregated by whether individuals were accessing care at Kaiser Permanente San Francisco or KPNC sites outside of San Francisco.

The statistical analysis was performed using Stata, version 17.0 (StataCorp LLC). A 2-sided *P* < .05 by *t* tests for continuous variables and χ^2^ tests for categorical variables was considered significant.

## Results

### Study Population and DoxyPEP Uptake

A total of 11 551 individuals received HIV PrEP at KPNC from November 1, 2022, through December 31, 2023 (mean [SD] age, 39.9 [12.1] years; 4.5% female, 95.1% male, 0.5% unknown sex; 0.4% American Indian or Alaska Native, 17.1% Asian, 6.1% Black or African American, 24.1% Hispanic, 0.7% Native Hawaiian or Pacific Islander, 44.7% White, 1.4% multiracial, and 5.6% unknown race and ethnicity) (Table 1). Of these individuals, 2253 (19.5%) were dispensed doxyPEP, with the quarterly number of individuals first dispensed doxyPEP increasing from 85 in Q4 2022 to 650 in Q3 2023, then decreasing to 605 in Q4 2023 (eFigure 1 in Supplement 1). The median length of follow-up after first being dispensed doxyPEP was 5.7 months (IQR, 2.9-8.4 months). Among individuals dispensed doxyPEP, 2228 (98.9%) were male, and 1096 (48.6%) had a bacterial STI diagnosis in the 12 months before starting doxyPEP (681 [30.2%] chlamydia, 664 [29.5%] gonorrhea, 120 [5.3%] syphilis) (Table 1). Compared with individuals not dispensed doxyPEP, doxyPEP recipients were older (mean [SD] age, 40.4 [10.8] vs 39.8 [12.4] years; *P* = .04) and had been in KPNC’s HIV PrEP program for longer (mean [SD], 4.2 [2.8] vs 3.4 [2.6] years; *P* < .001), and a higher proportion were commercially insured (2091 [92.8%] vs 8270 [88.9%]; *P* < .001). Race and ethnicity did not differ between individuals dispensed and not dispensed doxyPEP.

**Table 1.** Characteristics of HIV PrEP Users at KPNC Who Did and Did Not Initiate DoxyPEP From November 1, 2022, Through December 31, 2023

Characteristic	No. of individuals (%)			*P* value^a^
	All (N = 11 551)	Dispensed doxyPEP (n = 2253)	Not dispensed doxyPEP (n = 9298)	
Age, mean (SD) [range], y^b^	39.9 (12.1) [19-86]	40.4 (10.8) [19-84]	39.8 (12.4) [19-86]	.04
Age group, y^b^				
18-25	1051 (9.1)	104 (4.6)	947 (10.2)	<.001
26-35	3902 (33.8)	795 (35.3)	3107 (33.4)	
36-45	3320 (28.7)	735 (32.6)	2585 (27.8)	
>45	3278 (28.4)	619 (27.5)	2659 (28.6)	
Insurance type				
Commercial	10 361 (89.7)	2091 (92.8)	8270 (88.9)	<.001
Medicaid	777 (6.7)	107 (4.8)	670 (7.2)	
Medicare	393 (3.4)	52 (2.3)	341 (3.7)	
Other^c^	20 (0.2)	3 (0.1)	17 (0.2)	
Race and ethnicity				
American Indian or Alaska Native alone, non-Hispanic	48 (0.4)	8 (0.4)	40 (0.4)	>.99
Asian alone, non-Hispanic	1979 (17.1)	385 (17.1)	1594 (17.1)	
Black or African American alone, non-Hispanic	699 (6.1)	141 (6.3)	558 (6.0)	
Hispanic	2779 (24.1)	544 (24.1)	2235 (24.0)	
Native Hawaiian or Pacific Islander alone, non-Hispanic	79 (0.7)	14 (0.6)	65 (0.7)	
White alone, non-Hispanic	5164 (44.7)	1007 (44.7)	4157 (44.7)	
Multiracial, non-Hispanic	157 (1.4)	32 (1.4)	125 (1.3)	
Unknown, non-Hispanic	646 (5.6)	122 (5.4)	524 (5.6)	
Sex				
Male	10 982 (95.1)	2228 (98.9)	8754 (94.2)	<.001
Female	515 (4.5)	17 (0.8)	498 (5.4)	
Unknown	54 (0.5)	8 (0.4)	46 (0.5)	
Years since first recorded PrEP prescription at KPNC, mean (SD)^b^	3.6 (2.7)	4.2 (2.8)	3.4 (2.6)	<.001
Diagnosed with a bacterial STI in the past 12 mo^d^				
Any STI	2790 (24.2)	1096 (48.6)	1694 (18.2)	<.001
Chlamydia	1615 (14.0)	681 (30.2)	934 (10.0)	<.001
Gonorrhea	1596 (13.8)	664 (29.5)	932 (10.0)	<.001
Syphilis	316 (2.7)	120 (5.3)	196 (2.1)	<.001
Location of main site of care				
San Francisco	3462 (30.0)	873 (38.8)	2589 (27.8)	<.001
Outside San Francisco	8089 (70.0)	1380 (61.3)	6709 (72.2)	

Abbreviations: doxyPEP, doxycycline postexposure prophylaxis; KPNC, Kaiser Permanente Northern California; PrEP, preexposure prophylaxis; STI, sexually transmitted infection.

^a^ *P* values derived from *t* tests for continuous variables and χ^2^ tests for categorical variables.

^b^ Measured at the end of the study period (December 31, 2023) for both groups.

^c^ All KPNC members are insured, but the type of insurance could not be determined for this subset.

^d^ Measured as the 12 months before doxyPEP initiation among doxyPEP recipients and as the 12 months before November 1, 2022, for nonrecipients.

### DoxyPEP Dispensing

Of 2253 individuals dispensed doxyPEP at least once, 1969 (87.4%) received 30 doxycycline 100-mg pills in the first prescription; 114 (5.1%) received fewer and 170 (7.5%) received more than 30 pills (eTable 1 in Supplement 1). The median number of fills per recipient was 2 (IQR, 1-3). Among the 1175 (52.2%) individuals with multiple doxyPEP fills, the median number of days between fills was 52 (IQR, 28-89), and the median number of doses in possession per month was 6.5 (IQR, 4.3-10.6).

### STI Incidence Before and After Initiating DoxyPEP Among DoxyPEP Recipients

**Figure 1.**
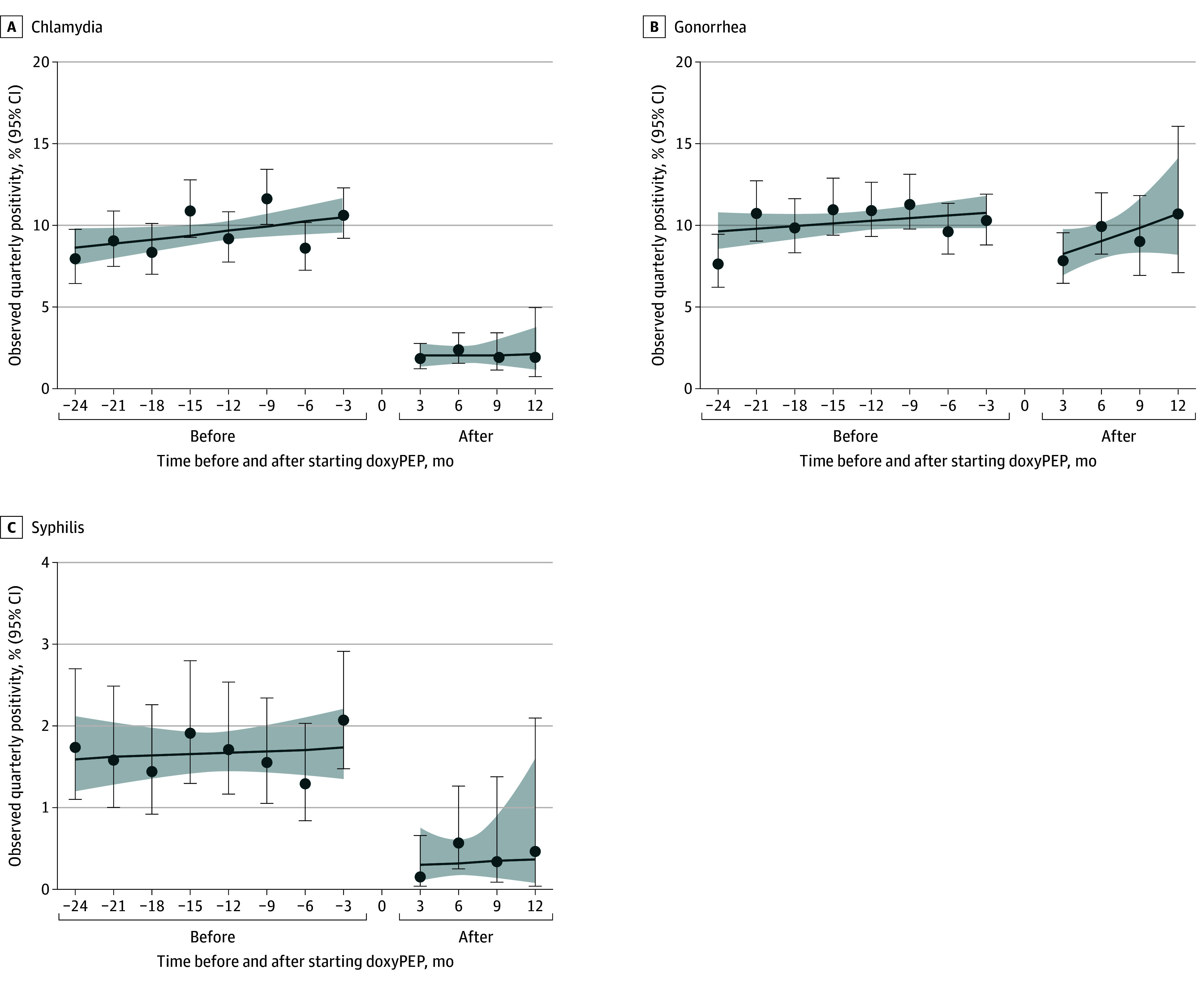
Quarterly Sexually Transmitted Infection Positivity From 24 Months Before to 12 Months After Starting Doxycycline Postexposure Prophylaxis (DoxyPEP) Among HIV Preexposure Prophylaxis Users Dispensed DoxyPEP at Least Once (n = 2253) Lines represent trends (mean quarterly positivity) for pre-doxyPEP and post-doxyPEP initiation; shaded areas, 95% CIs for trend. A baseline window from 90 days before to 14 days after starting doxyPEP was excluded. Chlamydia and gonorrhea positivity represent any anatomic site of infection. eFigure 2 in Supplement 1 shows quarterly chlamydia and gonorrhea positivity by anatomic site of infection.

Among doxyPEP recipients, quarterly chlamydia positivity in the 24 months before initiating doxyPEP ranged from 7.9% (95% CI, 6.4%-9.8%) to 11.6% (95% CI, 10.0%-13.4%) and in the 12 months after starting doxyPEP, from 1.8% (95% CI, 1.2%-2.7%) to 2.2% (95% CI, 1.5%-3.4%) (Figure 1A). Overall, mean chlamydia positivity declined from 9.6% (95% CI, 9.0%-10.3%) before starting doxyPEP to 2.0% (95% CI, 1.5%-2.6%) after starting doxyPEP (RR, 0.21; 95% CI, 0.16-0.27; *P* < .001) (Table 2). Statistically significant declines were observed separately for rectal (RR, 0.18; 95% CI, 0.13-0.24; *P* < .001), pharyngeal (RR, 0.16; 95% CI, 0.09-0.31; *P* < .001), and urethral (RR, 0.25; 95% CI, 0.16-0.40; *P* < .001) chlamydia positivity.

**Table 2.** Change in Quarterly STI Positivity Trends and Mean STI Positivity From Before to After Starting DoxyPEP Among HIV PrEP Users Dispensed DoxyPEP at Least Once (n = 2253)^a^

STI and anatomic site	Before starting doxyPEP^b^			After starting doxyPEP^c^			Comparison^d^	
	Mean positivity, % (95% CI)	Trend		Mean positivity, % (95% CI)	Trend			
		Mean change in positivity per quarter, % (95% CI)	*P* value		Mean change in positivity per quarter, % (95% CI)	*P* value	RR (95% CI)	*P* value
Chlamydia	9.6 (9.0 to 10.3)	3.0 (0.3 to 5.7)	.03	2.0 (1.5 to 2.6)	1.8 (−22.2 to 33.3)	.90	0.21 (0.16 to 0.27)	<.001
Rectal	7.4 (6.8 to 8.0)	3.2 (−0.1 to 6.5)	.055	1.3 (0.9 to 1.8)	1.3 (−28.1 to 42.7)	.94	0.18 (0.13 to 0.24)	<.001
Pharyngeal	2.0 (1.8 to 2.4)	5.1 (−1.0 to 11.6)	.11	0.3 (0.2 to 0.6)	−26.4 (−65.1 to 55.0)	.42	0.16 (0.09 to 0.31)	<.001
Urethral	2.6 (2.3 to 2.9)	1.9 (−3.2 to 7.3)	.47	0.7 (0.4 to 1.0)	26.0 (−19.4 to 96.7)	.31	0.25 (0.16 to 0.40)	<.001
Gonorrhea	10.2 (9.6 to 10.9)	1.7 (−0.9 to 4.3)	.21	9.0 (8.0 to 10.1)	9.4 (−3.3 to 23.8)	.16	0.88 (0.77 to 1.00)	.048
Rectal	6.5 (5.9 to 7.1)	−0.1 (−3.4 to 3.4)	.98	5.2 (4.4 to 6.2)	14.9 (−2.7 to 35.5)	.10	0.81 (0.67 to 0.97)	.02
Pharyngeal	7.0 (6.5 to 7.6)	1.8 (−1.4 to 5.1)	.27	6.4 (5.6 to 7.4)	1.1 (−13.1 to 17.6)	.89	0.91 (0.78 to 1.06)	.24
Urethral	2.1 (1.8 to 2.5)	−1.3 (−6.6 to 4.4)	.66	1.2 (0.9 to 1.7)	13.8 (−18.6 to 59.1)	.45	0.56 (0.40 to 0.79)	.001
Syphilis	1.7 (1.4 to 1.9)	1.1 (−5.1 to 7.8)	.73	0.3 (0.2 to 0.6)	7.1 (−44.3 to 106.2)	.84	0.20 (0.11 to 0.37)	<.001

Abbreviations: doxyPEP, doxycycline postexposure prophylaxis; PrEP, preexposure prophylaxis; RR, rate ratio; STI, sexually transmitted infection.

^a^ A baseline window from 90 days before to 14 days after starting doxyPEP was excluded.

^b^ The period before starting doxyPEP was the 24 months (8 quarters) prior to an individual’s baseline window.

^c^ The period after starting doxyPEP was 12 months (4 quarters) after an individual’s baseline window.

^d^ The RR represents the relative difference in mean quarterly positivity during the 12 months after starting doxyPEP compared with the mean quarterly STI positivity during the 24 months before starting doxyPEP.

Quarterly gonorrhea positivity ranged from 7.6% (95% CI, 6.2%-9.5%) to 11.3% (95% CI, 9.7%-13.1%) before starting doxyPEP and from 7.8% (95% CI, 6.4%-9.5%) to 10.7% (95% CI, 7.1%-16.1%) after starting doxyPEP (Figure 1B). Overall, mean gonorrhea positivity declined from 10.2% (95% CI, 9.6%-10.9%) before starting doxyPEP to 9.0% (95% CI, 8.0%-10.1%) after starting doxyPEP (RR, 0.88; 95% CI, 0.77-1.00; *P* = .048) (Table 2). Declines were statistically significant for rectal (RR, 0.81; 95% CI, 0.67-0.97; *P* = .02) and urethral (RR, 0.56; 95% CI, 0.40-0.79; *P* = .001) gonorrhea positivity but not for pharyngeal gonorrhea positivity (RR, 0.91; 95% CI, 0.78-1.06; *P* = .24) (Table 2).

Quarterly positivity for syphilis before starting doxyPEP ranged from 1.3% (95% CI, 0.8%-2.0%) to 2.1% (95% CI, 1.5%-2.9%) and after starting doxyPEP, from 0.2% (95% CI, 0.0%-0.7%) to 0.6% (95% CI, 0.3%-1.3%) (Figure 1C). Overall, mean syphilis positivity declined from 1.7% (95% CI, 1.4%-1.9%) before starting doxyPEP to 0.3% (95% CI, 0.2%-0.6%) after starting doxyPEP (RR, 0.20; 95% CI, 0.11-0.37; *P* < .001) (Table 2). eTable 2 in Supplement 1 provides the number of individuals contributing to STI positivity estimates in each quarter.

Sensitivity analyses among individuals starting doxyPEP before June 30, 2023, produced similar RRs for the reduction in STI positivity from 12 months before to 6 months after starting doxyPEP. No significant change was observed for pharyngeal gonorrhea positivity (eTable 3 in Supplement 1).

### STI Testing Before and After Starting DoxyPEP Among DoxyPEP Recipients

Among doxyPEP recipients, the mean proportion tested per quarter for chlamydia or gonorrhea and syphilis remained stable from 12 months before to 12 months after starting doxyPEP (eTable 4 in Supplement 1), while the mean number of tests per quarter declined slightly (eTable 5 in Supplement 1). Among the 987 individuals with at least 6 months of follow-up after starting doxyPEP, 923 (93.5%) returned for an STI test in the 6 months after starting doxyPEP.

### STI Incidence Before and After DoxyPEP Implementation in the Full Cohort

**Figure 2.**
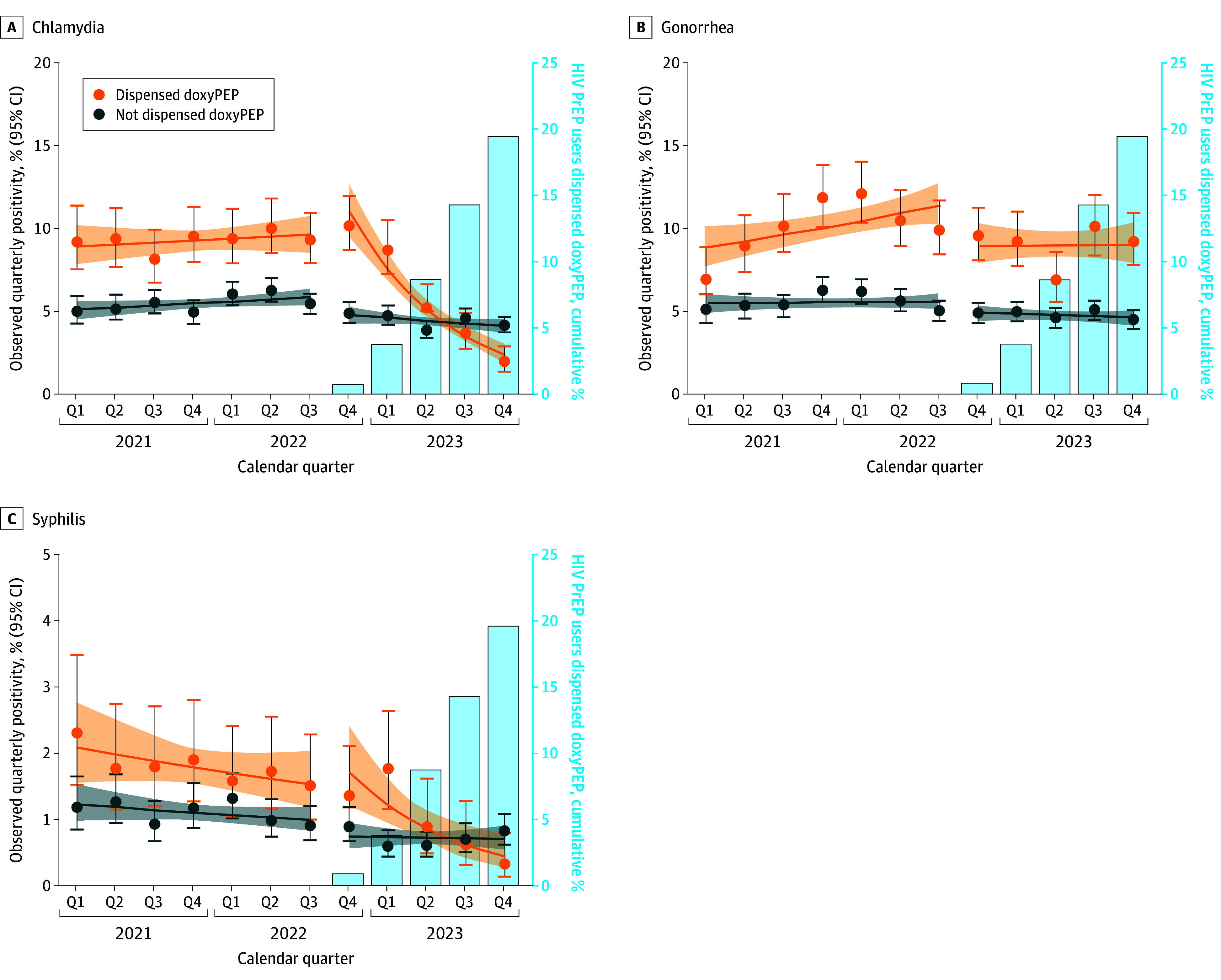
Trends in Quarterly Sexually Transmitted Infection Positivity From January 1, 2021, to December 31, 2023, Among HIV Preexposure Prophylaxis (PrEP) Users Dispensed and Not Dispensed Doxycycline Postexposure Prophylaxis (DoxyPEP) During the Study Period (N = 11 551) Solid lines are 95% CIs for trend in the pre-doxyPEP implementation period and post-doxyPEP implementation period. Chlamydia and gonorrhea positivity is at any anatomic site of infection.

During the pre-doxyPEP implementation period, quarterly chlamydia positivity was higher among individuals subsequently dispensed doxyPEP than those never dispensed doxyPEP, with no significant trend in either group (Figure 2A). During the post-doxyPEP implementation period, chlamydia positivity declined from 10.2% (95% CI, 8.7%-12.0%) in Q4 2022 to 2.0% (95% CI, 1.4%-2.9%) in Q4 2023 (mean change per quarter, −31.8%; 95% CI, −36.7% to −26.4%; *P* < .001) in the doxyPEP group as the proportion dispensed doxyPEP increased, while it remained stable among individuals never dispensed doxyPEP (Table 3).

**Table 3.** Quarterly Trends in STI Positivity From January 1, 2021, to December 31, 2023, Among HIV PrEP Users at KPNC Dispensed and Not Dispensed DoxyPEP at Least Once

STI	Before doxyPEP implementation		After doxyPEP implementation	
	Mean % change in positivity per quarter (95% CI)	*P* value	Mean % change in positivity per quarter (95% CI)	*P* value
Chlamydia				
Dispensed doxyPEP	1.2 (−2.2 to 4.8)	.49	−31.8 (−36.7 to −26.4)	<.001
Not dispensed doxyPEP	2.4 (−0.2 to 5.0)	.07	−3.3 (−7.1 to 0.7)	.10
Gonorrhea				
Dispensed doxyPEP	4.3 (0.9 to 7.8)	.01	0.1 (−5.3 to 5.8)	.98
Not dispensed doxyPEP	0.4 (−2.1 to 3.0)	.75	−1.6 (−5.3 to 2.3)	.42
Syphilis				
Dispensed doxyPEP	−5.0 (−12.3 to 2.8)	.20	−29.3 (−41.4 to −14.8)	<.001
Not dispensed doxyPEP	−3.6 (−9.0 to 2.0)	.21	−0.9 (−10.4 to 9.6)	.86

Abbreviations: doxyPEP, doxycycline postexposure prophylaxis; KPNC, Kaiser Permanente Northern California; PrEP, preexposure prophylaxis; STI, sexually transmitted infection.

During the pre-doxyPEP implementation period, quarterly gonorrhea positivity was higher and increased slightly from 6.9% (95% CI, 5.4%-8.8%) in Q1 2021 to 9.9% (95% CI, 8.4%-11.6%) in Q3 2022 among individuals who subsequently received doxyPEP (mean change per quarter, 4.3%; 95% CI, 0.9%-7.8%), while remaining stable among those never dispensed doxyPEP (Figure 2B). During the post-doxyPEP implementation period, quarterly gonorrhea positivity remained higher in the doxyPEP group, with no significant trend in either group.

During the pre-doxyPEP implementation period, quarterly syphilis positivity was higher among individuals subsequently dispensed doxyPEP than those never dispensed doxyPEP, with no significant trend in either group (Figure 2C). During the post-doxyPEP implementation period, syphilis positivity declined from 1.3% (95% CI, 0.8%-2.1%) in Q4 2022 to 0.3% (95% CI, 0.1%-0.8%) in Q4 2023 (mean change per quarter, –29.3%; 95% CI, −41.4% to −14.8%; *P* < .001) in the doxyPEP group as the proportion dispensed doxyPEP increased, while remaining stable among those never dispensed doxyPEP.

Compared with those never dispensed doxyPEP, doxyPEP recipients had lower chlamydia positivity (RR, 0.45; 95% CI, 0.35-0.59; *P* < .001) and syphilis positivity (RR, 0.49; 95% CI, 0.26-0.92; *P* = .03) and higher gonorrhea positivity (RR, 1.97; 95% CI, 1.72-2.25; *P* < .001) after initiating doxyPEP. Similar results were found after adjusting for age, race and ethnicity, and type of insurance (eTable 6 in Supplement 1).

Overall, 70.0% of all HIV PrEP users were accessing care outside of San Francisco. Among all HIV PrEP users, quarterly chlamydia and syphilis positivity in the post-doxyPEP implementation period declined at faster rates in San Francisco than outside of San Francisco (eTable 7 in Supplement 1).

## Discussion

In this large cohort study of individuals accessing HIV PrEP in an integrated health care system in northern California, 1 in 5 received doxyPEP during the first year of availability. Receipt of doxyPEP was associated with substantial declines in chlamydia and syphilis incidence and with moderate declines in urethral and rectal gonorrhea but not pharyngeal gonorrhea. In contrast, STI positivity was stable in the post-doxyPEP implementation period among individuals not dispensed doxyPEP. Our data represent early estimates of the association between receipt of doxyPEP and decreases in STI incidence in clinical practice and highlight the potential benefits of doxyPEP implementation for reducing STIs in sexual networks of people using HIV PrEP.

The declines we observed in quarterly STI positivity among individuals starting doxyPEP mirror those observed in clinical trials. Two large randomized clinical trials of doxyPEP among cisgender gay and bisexual men and transgender women who were either living with HIV or using HIV PrEP reported that incidence decreased by 74% to 88% for chlamydia and 73% to 87% for syphilis.^1,2^ Evidence for the impact of doxyPEP use on gonorrhea in trials was mixed, with 1 trial showing no impact^1^ and another showing decreases between 55% and 57%.^2^ Varying protection against gonorrhea may reflect varying background levels of tetracycline resistance; for example, the prevalence of tetracycline resistance of gonorrhea in France is estimated to be greater than 50% compared with 20.6% in the US.^9^

Although we could not infer causation in this observational study, it is noteworthy that rates of chlamydia and syphilis among individuals dispensed doxyPEP rapidly declined while remaining stable among those not dispensed doxyPEP. By the end of the study period, chlamydia and syphilis positivity were lower among doxyPEP recipients than nonrecipients, despite being higher among doxyPEP recipients than nonrecipients prior to doxyPEP implementation. We also observed greater declines in STI positivity following doxyPEP implementation among individuals accessing care in San Francisco. As doxyPEP was scaled up at several non-KPNC sites in San Francisco during the same period^10^ and declines in syphilis and rectal chlamydia and gonorrhea were recently observed among men in San Francisco following issuance of doxyPEP guidelines,^11^ our findings may reflect benefits of wider doxyPEP implementation for interrupting STI transmission in sexual networks of people using HIV PrEP.

We observed rapid uptake of doxyPEP in this cohort of HIV PrEP users, consistent with a recent nationwide survey of primarily gay and bisexual men that found near-universal interest in the use of antibiotic STI prophylaxis.^12^ Although we did not observe differences in uptake by race or ethnicity, commercial insurance was more prevalent among individuals dispensed doxyPEP, suggesting a potential role of health care access or other social determinants of health in doxyPEP use and highlighting the need for efforts to ensure equitable implementation.

Although declines in STI screening may offset the STI prevention benefits of doxyPEP, we observed largely stable testing rates after starting doxyPEP, with only slight declines after doxyPEP initiation and nearly all doxyPEP recipients returning for an STI test within 6 months of initiation. Centers for Disease Control and Prevention guidelines now recommend that doxyPEP users be screened for chlamydia, gonorrhea, and syphilis every 3 to 6 months.^5^ Continued monitoring of STI testing and incidence over longer periods of doxyPEP use, among later adopters of doxyPEP, and among doxyPEP users not engaged in HIV PrEP care will be important for evaluating and guiding doxyPEP implementation.

Scientists^13,14^ and communities of people who could benefit from doxyPEP^12^ have expressed concern that doxyPEP could increase antimicrobial resistance in STIs and other infections through increased selection of resistant strains. Consistent with clinical trials,^2^ we found that reductions in pharyngeal gonorrhea were less pronounced than for rectal and urethral gonorrhea, a pattern that may reflect poor concentration of doxycycline in pharyngeal mucosa^15^ and may pose an increased risk of resistance acquisition through horizontal transfer of resistance genes from commensal *Neisseria* species.^16^ Monitoring for resistance in gonorrhea and other infections (eg, *Staphylococcus aureus*) among people using doxyPEP will be important as doxyPEP is scaled up.

### Strengths and Limitations

Our study has several strengths. First, we used pharmacy dispensing data, which are not typically available in electronic health records and provide a more robust measure of medication use than prescription data alone.^17^ Second, our study setting covered a large geographic area, allowing us to explore variations in STI trends by region. Third, long-term engagement in HIV PrEP care provided up to 2 years of STI data before individuals started doxyPEP. Finally, inclusion of individuals not dispensed doxyPEP allowed us to compare trends in STIs by receipt of doxyPEP.

Our study also has several limitations. First, early adopters of doxyPEP in our cohort of HIV PrEP users are not representative of all people who may benefit from or use doxyPEP. For example, less than 7% of our cohort was Black, whereas the rate of primary and secondary syphilis is highest among Black men, with a rate of 68.3 per 100 000 compared with 25.2 per 100 000 among all men in 2021.^9^ Second, data were not available on gender identity or sexual orientation; however, individuals using HIV PrEP at KPNC have been previously described as primarily gay and bisexual men.^18^ Third, we relied on laboratory results to detect new cases of syphilis and may have missed some cases; however, our definition of incident syphilis was consistent across pre- and post-doxyPEP implementation periods, minimizing potential bias. Fourth, we were unable to measure changes in sexual behavior (eg, in response to the mpox outbreak) that may have influenced STI incidence during the doxyPEP implementation period. However, STI positivity did not decline among nonrecipients of doxyPEP, suggesting that any changes in sexual behavior did not have a measurable impact. Fifth, as STI treatment data were not extracted, we could not ensure that every STI was treated and each positive test represented a new infection; however, the KPNC HIV PrEP program is highly experienced in managing STIs and follows national STI treatment guidelines. Finally, due to the observational nature of our study, we were not able to estimate causal effects of doxyPEP on STI incidence, and there are several contextual considerations when interpreting our results. As starting doxyPEP is associated with STI testing and treatment of prevalent STIs, most doxyPEP recipients would have been STI-free at the start of the period after doxyPEP initiation, whereas there was no similarly systematic testing at the start of the period before doxyPEP initiation. Furthermore, as starting doxyPEP is likely associated with being recently diagnosed with an STI, the period immediately before starting doxyPEP may reflect a period of increased STI risk. However, the attenuated reduction in gonorrhea incidence we observed suggests that these factors likely had a limited influence on our incidence reduction estimates. Future analyses should use causal methods, such as target trial emulation,^19^ to address biases associated with observational data.

## Conclusions

In this cohort study, among individuals accessing HIV PrEP, receipt of doxyPEP was associated with substantial declines in chlamydia and syphilis incidence and moderate, site-specific declines in gonorrhea incidence during the first year of doxyPEP availability. DoxyPEP may offer substantial benefits for reducing population-level STI transmission with broader implementation.

## Notes

Supplement 1. **eMethods.** Information on Syphilis Determination Algorithm
eReferences
**eFigure 1.** Quarterly Number of HIV PrEP Users Initiating DoxyPEP During the Study Period **eTable 1.** Number of Pills Dispensed, Number of DoxyPEP Fills per Person, and Available DoxyPEP Doses During Follow-Up **eTable 2.** Number of Individuals Contributing to Quarterly STI Positivity Estimates From 24 Months Before Starting DoxyPEP to 12 Months After Starting doxyPEP **eFigure 2.** Quarterly Chlamydia and Gonorrhea Positivity From Before to After Starting doxyPEP Among DoxyPEP Recipients (n = 2253), by Anatomical Site of Infection **eFigure 3.** Quarterly Proportion of DoxyPEP Recipients Tested for Chlamydia/Gonorrhea and Syphilis Among doxyPEP Users (n = 2253), Before and After Starting DoxyPEP **eTable 3.** Sensitivity Analysis Restricted to Individuals First Dispensed doxyPEP Before June 30, 2023, and Comparing First 6 Months After Starting doxyPEP With 12 Months Before Starting DoxyPEP **eTable 4.** Quarterly Trends in Proportion Tested and Change in Proportion Tested From Before to After Starting DoxyPEP Among doxyPEP Users (n = 2253) **eTable 5.** Mean Number of Tests per Quarter Among doxyPEP Users Before and After Starting DoxyPEP **eFigure 4.** Quarterly STI Positivity from January 1, 2021, to December 31, 2023, Among HIV PrEP Users Receiving Care in and Outside of San Francisco (N = 11 551) **eTable 6.** Comparison of STI Positivity Among DoxyPEP Recipients After doxyPEP Initiation and STI Positivity Among Non-doxyPEP Recipients During the Post-doxyPEP Implementation Phase (N = 11 551) **eTable 7.** Trends in Quarterly STI Positivity Before and After doxyPEP Implementation Among HIV PrEP Users Receiving Care in and outside of San Francisco (N = 11 551)

Supplement 2.
Data Sharing Statement
