# Supplementary material for: Doxycycline Postexposure Prophylaxis and Bacterial Sexually Transmitted Infections Among Individuals Using HIV Preexposure Prophylaxis
Source: JAMA Intern Med. 2025 Jan 6;185(3):273–81. doi: 10.1001/jamainternmed.2024.7186 (PMC11877173; doi:10.1001/jamainternmed.2024.7186)
Supplement: Supplement 1. — eMethods. Information on Syphilis Determination Algorithm eReferences eFigure 1. Quarterly Number of HIV PrEP Users Initiating DoxyPEP During the Study Period eTable 1. Number of Pills Dispensed, Number of DoxyPEP Fills per Person, and Available DoxyPEP Doses During Follow-Up eTable 2. Number of Individuals Contributing to Quarterly STI Positivity Estimates From 24 Months Before Starting DoxyPEP to 12 Months After Starting doxyPEP eFigure 2. Quarterly Chlamydia and Gonorrhea Positivity From Before to After Starting doxyPEP Among DoxyPEP Recipients (n = 2253), by Anatomical Site of Infection eFigure 3. Quarterly Proportion of DoxyPEP Recipients Tested for Chlamydia/Gonorrhea and Syphilis Among doxyPEP Users (n = 2253), Before and After Starting DoxyPEP eTable 3. Sensitivity Analysis Restricted to Individuals First Dispensed doxyPEP Before June 30, 2023, and Comparing First 6 Months After Starting doxyPEP With 12 Months Before Starting DoxyPEP eTable 4. Quarterly Trends in Proportion Tested and Change in Proportion Tested From Before to After Starting DoxyPEP Among doxyPEP Users (n = 2253) eTable 5. Mean Number of Tests per Quarter Among doxyPEP Users Before and After Starting DoxyPEP eFigure 4. Quarterly STI Positivity from January 1, 2021, to December 31, 2023, Among HIV PrEP Users Receiving Care in and Outside of San Francisco (N = 11 551) eTable 6. Comparison of STI Positivity Among DoxyPEP Recipients After doxyPEP Initiation and STI Positivity Among Non-doxyPEP Recipients During the Post-doxyPEP Implementation Phase (N = 11 551) eTable 7. Trends in Quarterly STI Positivity Before and After doxyPEP Implementation Among HIV PrEP Users Receiving Care in and outside of San Francisco (N = 11 551) [file jamainternmed-e247186-s001.pdf]

Supplemental Online Content

Traeger MW, Leyden WA, Volk JE, et al. Doxycycline postexposure prophylaxis and bacterial sexually transmitted infections among individuals using HIV pre-exposure prophylaxis. *JAMA Intern Med*. Published online January 6, 2025. doi:10.1001/jamainternmed.2024.7186

eMethods. Information on Syphilis Determination Algorithm

eReferences

eFigure 1. Quarterly Number of HIV PrEP Users Initiating doxyPEP During the Study Period

eTable 1. Number of Pills Dispensed, Number of doxyPEP Fills per Person, and Available doxyPEP Doses During Follow-Up

eTable 2. Number of Individuals Contributing to Quarterly STI Positivity Estimates From 24 Months Before Starting doxyPEP to 12 Months After Starting doxyPEP

eFigure 2. Quarterly Chlamydia and Gonorrhea Positivity From Before to After Starting doxyPEP Among doxyPEP Recipients (n = 2253), by Anatomical Site of Infection

eFigure 3. Quarterly Proportion of doxyPEP Recipients Tested for Chlamydia/Gonorrhea and Syphilis Among doxyPEP Users (n = 2253), Before and After Starting doxyPEP

eTable 3. Sensitivity Analysis Restricted to Individuals First Dispensed doxyPEP Before June 30, 2023, and Comparing First 6 Months After Starting doxyPEP With 12 Months Before Starting doxyPEP

eTable 4. Quarterly Trends in Proportion Tested and Change in Proportion Tested From Before to After Starting doxyPEP Among doxyPEP Users (n = 2253)

eTable 5. Mean Number of Tests per Quarter Among doxyPEP Users Before and After Starting doxyPEP

eFigure 4. Quarterly STI Positivity from January 1, 2021, to December 31, 2023, Among HIV PrEP Users Receiving Care in and Outside of San Francisco (N = 11 551)

eTable 6. Comparison of STI Positivity Among doxyPEP Recipients After doxyPEP Initiation and STI Positivity Among Non-doxyPEP Recipients During the Post-doxyPEP Implementation Phase (N = 11 551)

eTable 7. Trends in Quarterly STI Positivity Before and After doxyPEP Implementation Among HIV PrEP Users Receiving Care in and outside of San Francisco (N = 11 551)

This supplemental material has been provided by the authors to give readers additional information about their work.

© 2025 American Medical Association. All rights reserved.

eMethods. Information on Syphilis Determination Algorithm

New cases of syphilis were determined from laboratory results based on the reverse sequence testing algorithm.[1] Serologic testing for syphilis was conducted using a treponemal IgG and IgM antibody test followed by a confirmatory rapid plasma regain (RPR) test, with treponema pallidum particle agglutination confirmatory testing for discrepant results. For individuals with a history of reactive RPR, an increase in RPR titre of two dilutions was used to determine reinfection.[2]

eReferences

1. Papp JR, Park IU, Fakile Y, Pereira L, Pillay A, Bolan GA. CDC Laboratory Recommendations for Syphilis Testing, United States, 2024. *MMWR Recomm Rep*. Feb 8 2024;73(1):1-32. doi:10.15585/mmwr.rr7301a1
2. Menza TW, Levine K, Grasso C, Mayer K. Evaluation of 4 Algorithms to Identify Incident Syphilis Among HIV-Positive Men Who Have Sex With Men Engaged in Primary Care. *Sexually transmitted diseases*. Apr 2019;46(4):e38-e41. doi:10.1097/OLQ.0000000000000938

© 2025 American Medical Association. All rights reserved.

eFigure 1. Quarterly Number of HIV PrEP Users Initiating doxyPEP During the Study Period

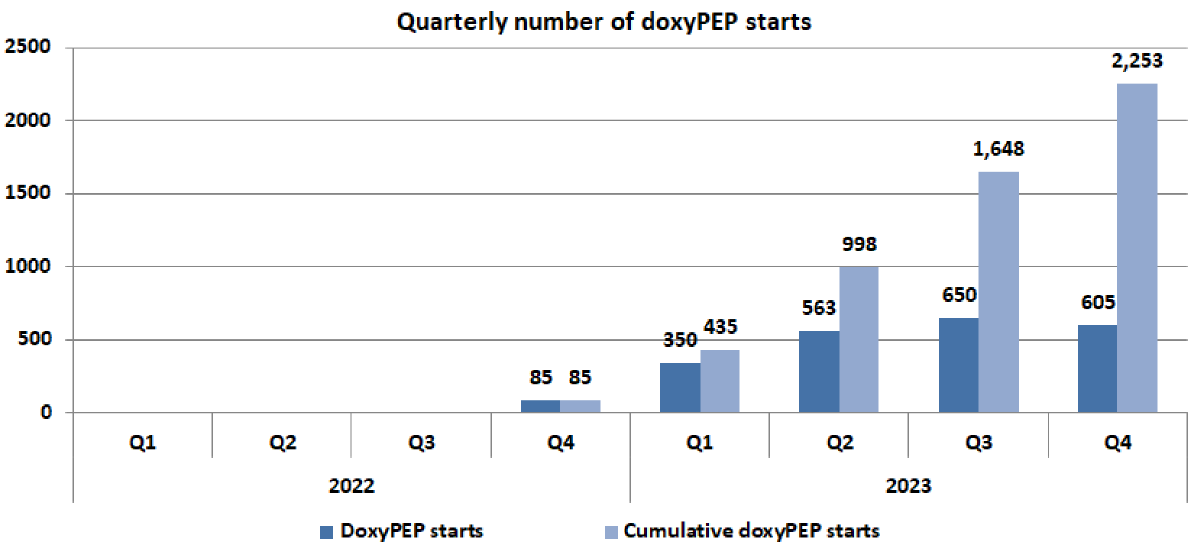

© 2025 American Medical Association. All rights reserved.

eTable 1. Number of Pills Dispensed, Number of doxyPEP Fills per Person, and Available doxyPEP Doses During Follow-Up

**Number of people dispensed doxyPEP at least once, n (% of all PrEP users)**		**2,253 (19.5%)**
**Number of pills in first fill**		
	**Mean (SD)**	32.0 (11.0)
	**Median (IQR, range)**	30 (30-30, 6-200)
**Number of pills in first fill, n (% of doxyPEP users)**		
	**<20**	35 (1.6)
	**20-29**	79 (3.5)
	**30**	1,969 (87.4)
	**31-59**	9 (0.4)
	**60**	142 (6.3)
	**>60**	19 (0.8)
**Number of doxyPEP fills per person**		
	**Mean (SD)**	2.3 (1.9)
	**Median (IQR)**	2 (1-3)
**Days between doxyPEP fills among those with multiple fills (n=1,175)**		
	**Mean (SD)**	66.2 (52.2)
	**Median (IQR)**	52 (28-89)
**Number of doxyPEP doses in possession per month during follow-up**		
	**Mean (SD)**	8.9 (8.3)
	**Median (IQR)**	6.5 (4.3 – 10.6)

Footnote: Number of doxyPEP doses in possession per month was calculated as the total number of doses (2 doxycycline 100mg pills) dispensed from the first to the last doxyPEP fill (excluding doses dispensed in the last fill) divided by the number of 30-day blocks of follow-up time during that window, with the analysis restricted to the subset of individuals with multiple doxyPEP fills.

© 2025 American Medical Association. All rights reserved.

eTable 2. Number of Individuals Contributing to Quarterly STI Positivity Estimates From 24 Months Before Starting doxyPEP to 12 Months After Starting doxyPEP

	Chlamydia			Gonorrhea			Syphilis		
**Quarter**	N. positive	N. tested	Positivity	N. positive	N. tested	Positivity	N. positive	N. tested	Positivity
**-24 m**	88	1,112	7.9%	85	1,112	7.6%	19	1,099	1.7%
**-21 m**	109	1,206	9.0%	129	1,206	10.7%	19	1,196	1.6%
**-18 m**	112	1,341	8.4%	132	1,341	9.8%	19	1,314	1.4%
**-15 m**	149	1,368	10.9%	150	1,368	11.0%	26	1,358	1.9%
**-12 m**	135	1,475	9.2%	160	1,475	10.8%	25	1,451	1.7%
**-9 m**	178	1,540	11.6%	174	1,540	11.3%	24	1,528	1.6%
**-6 m**	132	1,538	8.6%	148	1,538	9.6%	20	1,526	1.3%
**-3 m**	174	1,641	10.6%	168	1,641	10.2%	34	1,632	2.1%
**3m**	22	1,217	1.8%	95	1,217	7.8%	2	1,191	0.2%
**6 m**	24	1,068	2.2%	106	1,068	9.9%	6	1,054	0.6%
**9 m**	11	587	1.9%	53	587	9.0%	2	577	0.3%
**12 m**	4	215	1.9%	23	215	10.7%	1	216	0.5%

A baseline window from 90 days before to 14 days after starting doxyPEP was excluded. Chlamydia and gonorrhea positivity represent positivity at any anatomical site of infection.

© 2025 American Medical Association. All rights reserved.

eFigure 2. Quarterly Chlamydia and Gonorrhea Positivity From Before to After Starting doxyPEP Among doxyPEP Recipients (n = 2253), by Anatomical Site of Infection

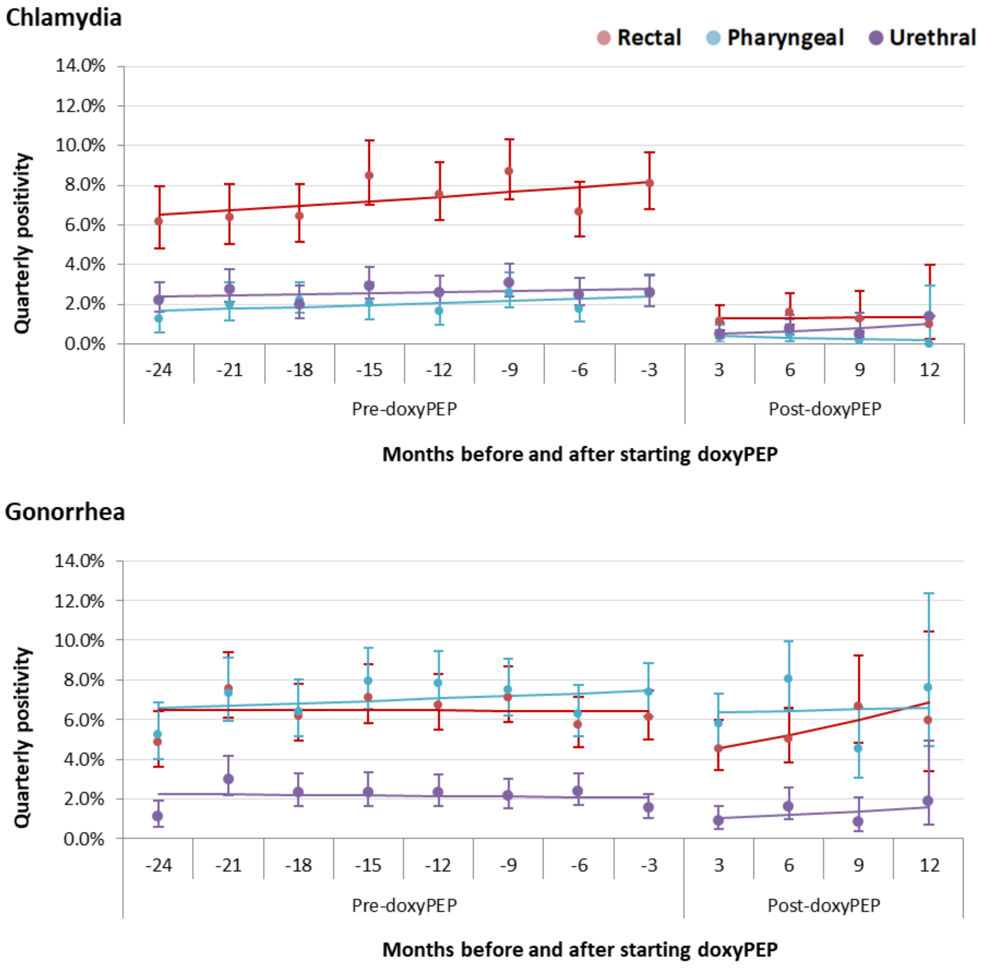

Trend lines represent mean quarterly positivity calculated from Poisson regression models for the periods before and after doxyPEP initiation separately. A baseline window from 90 days before to 14 days after the first doxyPEP dispensing date was excluded. Mean positivity and rate ratios representing change in positivity from before to after doxyPEP initiation are shown in Table 3.

© 2025 American Medical Association. All rights reserved.

eFigure 3. Quarterly Proportion of doxyPEP Recipients Tested for Chlamydia/Gonorrhea and Syphilis Among doxyPEP Users (n = 2253), Before and After Starting doxyPEP

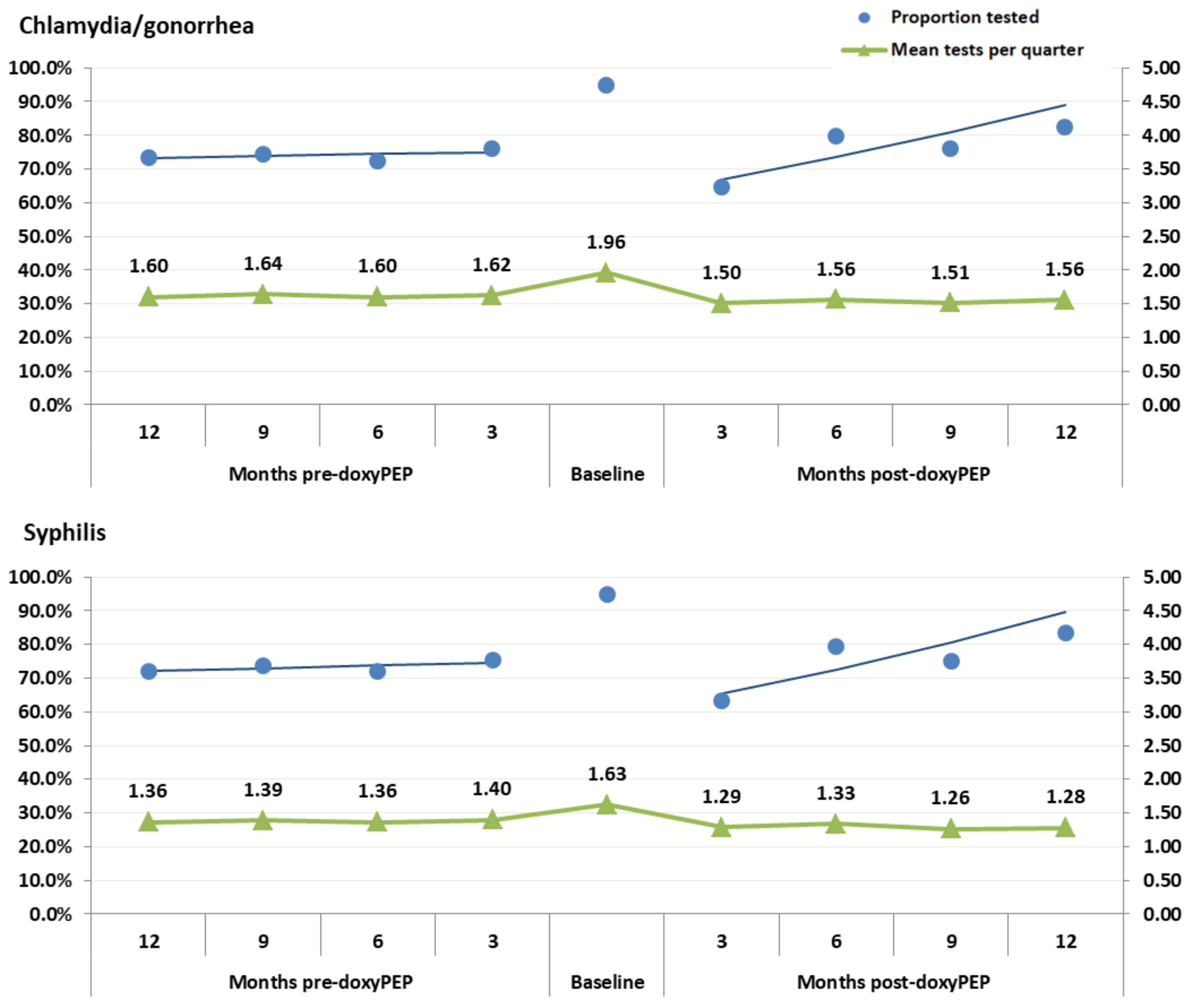

© 2025 American Medical Association. All rights reserved.

eTable 3. Sensitivity Analysis Restricted to Individuals First Dispensed doxyPEP Before June 30, 2023, and Comparing First 6 Months After Starting doxyPEP With 12 Months Before Starting doxyPEP

	Mean quarterly STI positivity before starting doxyPEP (95% CI)	Mean quarterly STI positivity after starting doxyPEP (95% CI)	RR (CI)	*P*-value
**Chlamydia**	11.1% (9.96-12.4)	2.1% (1.5-3.1)	0.19 (0.13-0.28)	<0.001
**Rectal**	6.2% (5.5-7.0)	1.1% (0.7-1.7)	0.18 (0.11-0.29)	<0.001
**Pharyngeal**	2.5% (1.9-3.2)	0.4% (0.1-0.8)	0.14 (0.06-0.35)	<0.001
**Urethral**	2.6% (2.1-3.3)	0.7% (0.4-1.3)	0.26 (0.14-0.51)	<0.001
**Gonorrhea**	12.6% (11.4-14.1)	8.5% (7.1-10.1)	0.67 (0.55-0.81)	<0.001
**Rectal**	8.0% (7.0-9.2)	4.2% (3.2-5.5)	0.52 (0.39-0.70)	<0.001
**Pharyngeal**	8.4% (7.3-9.6)	7.1% (5.8-8.6)	0.85 (0.67-1.06)	0.152
**Urethral**	2.5% (2.0-3.2)	1.1% (0.7-1.9)	0.45 (0.26-0.75)	0.002
**Syphilis**	2.0% (1.5-2.6)	0.3% (0.1-0.8)	0.18 (0.07-0.43)	<0.001

CI = confidence interval, RR = rate ratio.
Before doxyPEP period was the 12 months (4 quarters) prior to an individual's baseline window.
After doxyPEP period was 6 months (2 quarters) after an individual's baseline window.

© 2025 American Medical Association. All rights reserved.

eTable 4. Quarterly Trends in Proportion Tested and Change in Proportion Tested From Before to After Starting doxyPEP Among doxyPEP Users (n = 2253)

	Before starting doxyPEP			After starting doxyPEP			Comparison	
	Mean proportion tested per quarter	% change in proportion tested per quarter (CI)	p-value	Mean proportion tested per quarter	% change in proportion tested per quarter (CI)	p-value	RR (CI)	*P*-value
**Chlamydia /gonorrhea**	74.1%	0.8 (-1.5 – 3.1)	0.493	76.1%	4.8 (-0.6 – 10.5)	0.081	1.03 (0.98 – 1.08)	0.297
**Syphilis**	73.4%	1.1 (-1.2 – 3.5)	0.344	75.4%	5.4 (-1.0 – 11.2)	0.510	1.03 (0.98 – 1.08)	0.281

CI = confidence interval, RR = rate ratio.
Before doxyPEP period was the 24 months (8 quarters) prior to an individual's baseline window.
After doxyPEP period was 12 months (4 quarters) after an individual's baseline window.

eTable 5. Mean Number of Tests per Quarter Among doxyPEP Users Before and After Starting doxyPEP

	Mean tests per person per quarter		
	Before starting doxyPEP	After starting doxyPEP	*P*-value
**Chlamydia / gonorrhea**	1.62	1.56	0.0125
**Syphilis**	1.37	1.32	<0.001

Before doxyPEP period was the 24 months (8 quarters) prior to an individual's baseline window.
After doxyPEP period was 12 months (4 quarters) after an individual's baseline window.

© 2025 American Medical Association. All rights reserved.

eFigure 4. Quarterly STI Positivity from January 1, 2021, to December 31, 2023, Among HIV PrEP Users Receiving Care in and Outside of San Francisco (N = 11 551)

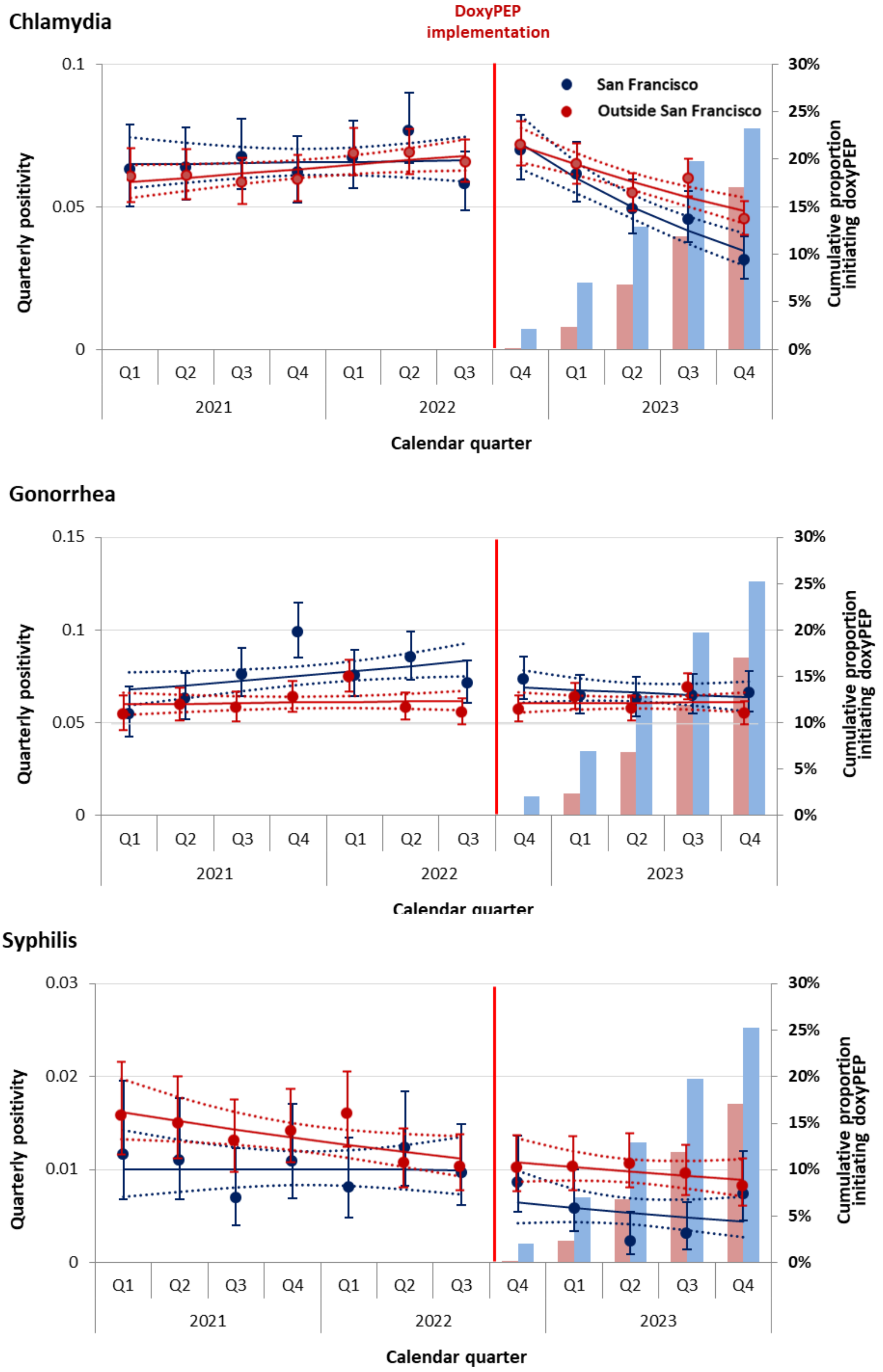

Blue (San Francisco) and red (outside San Francisco) dots with vertical bars represent observed quarterly positivity and 95% confidence intervals. Dotted lines are 95% confidence intervals for estimated trend in each period. Grey columns represent cumulative proportion of cohort ever dispensed doxyPEP.

© 2025 American Medical Association. All rights reserved.

eTable 6. Comparison of STI Positivity Among doxyPEP Recipients After doxyPEP Initiation and STI Positivity Among Non-doxyPEP Recipients During the Post-doxyPEP Implementation Phase (N = 11 551)

	Mean quarterly positivity among non-doxyPEP recipients (95% CI)	Mean quarterly positivity among doxyPEP recipients after doxyPEP initiation (95% CI)	Unadjusted RR (95% CI)	*P*-value	Adjusted RR* (95% CI)	*P*-value
Chlamydia	4.6% (4.3 – 4.9%)	2.1% (1.6 – 2.7)	0.45 (0.35 – 0.59)	<0.001	0.51 (0.39 – 0.66)	<0.001
Gonorrhea	4.7% (4.5 – 0.50)	9.3% (8.3 – 10.5)	1.97 (1.72 – 2.25)	<0.001	2.08 (1.81 – 2.39)	<0.001
Syphilis	0.7% (0.6 – 0.8)	0.3% (0.2 – 0.6)	0.49 (0.26 – 0.92)	0.026	0.52 (0.27 – 0.99)	0.045

*Adjusted for calendar quarter, age, race, ethnicity, and type of insurance.
CI=confidence interval, RR=rate ratio

© 2025 American Medical Association. All rights reserved.

eTable 7. Trends in Quarterly STI Positivity Before and After doxyPEP Implementation Among HIV PrEP Users Receiving Care in and outside of San Francisco (N = 11 551)

	Pre-doxyPEP implementation		Post-doxyPEP implementation	
	Mean % change in positivity per quarter (CI)	p-value	Mean % change in positivity per quarter (CI)	p-value
**Chlamydia**				
**All PrEP users**	1.8 (-0.3 – 3.9)	0.089	**-11.2 (-14 – -8.5)**	**<0.001**
**San Francisco**	0.4 (-3.2 – 4)	0.845	**-16.7 (-21.6 – -11.6)**	**<0.001**
**Outside San Francisco**	2.5 (0 – 5.1)	0.052	**-9.2 (-12.4 – -5.8)**	**<0.001**
**Gonorrhea**				
**All PrEP users**	1.6 (-0.5 – 3.6)	0.131	-0.7 (-3.5 – 2.3)	0.655
**San Francisco**	**3.6 (0.2 – 7.1)**	**0.039**	-2.0 (-7 – 3.2)	0.438
**Outside San Francisco**	0.5 (-2 – 3.1)	0.697	0.0 (-3.5 – 3.7)	0.987
**Syphilis**				
**All PrEP users**	-4.3 (-8.7 – 0.2)	0.061	**-8.9 (-16.6 – -0.6)**	**0.036**
**San Francisco**	-0.1 (-8.8 – 9.5)	0.989	-15.7 (-31.5 – 3.8)	0.108
**Outside San Francisco**	**-5.9 (-10.8 – -0.7)**	**0.027**	-7.5 (-16 – 1.9)	0.113

CI = confidence interval
Pre-doxyPEP implementation period = January 1, 2021 – September 30, 2022
Post-doxyPEP implementation period = October 1, 2022 – December 31, 2023

© 2025 American Medical Association. All rights reserved.
