# Supplementary material for: Doxycycline Postexposure Prophylaxis and Bacterial Sexually Transmitted Infections Among Individuals Using HIV Preexposure Prophylaxis
Source: JAMA Intern Med. 2025 Jan 6;185(3):273–81. doi: 10.1001/jamainternmed.2024.7186 (PMC11877173; doi:10.1001/jamainternmed.2024.7186)
Supplement: Supplement 2. — Data Sharing Statement [file jamainternmed-e247186-s002.pdf]

Data Sharing Statement

Traeger. Doxycycline Postexposure Prophylaxis and Bacterial Sexually Transmitted Infections Among Individuals Using HIV Preexposure Prophylaxis. *JAMA Intern Med*. Published January 06, 2025. doi:10.1001/jamainternmed.2024.7186

Data
Data available: No
